# The mining of toxin-like polypeptides from EST database by single residue distribution analysis

**DOI:** 10.1186/1471-2164-12-88

**Published:** 2011-01-31

**Authors:** Sergey Kozlov, Eugene Grishin

**Affiliations:** 1 Shemyakin-Ovchinnikov Institute of Bioorganic Chemistry, Russian Academy of Sciences ul. Miklukho-Maklaya, 16/10, 117997, Moscow, Russia

## Abstract

**Background:**

Novel high throughput sequencing technologies require permanent development of bioinformatics data processing methods. Among them, rapid and reliable identification of encoded proteins plays a pivotal role. To search for particular protein families, the amino acid sequence motifs suitable for selective screening of nucleotide sequence databases may be used. In this work, we suggest a novel method for simplified representation of protein amino acid sequences named Single Residue Distribution Analysis, which is applicable both for homology search and database screening.

**Results:**

Using the procedure developed, a search for amino acid sequence motifs in sea anemone polypeptides was performed, and 14 different motifs with broad and low specificity were discriminated. The adequacy of motifs for mining toxin-like sequences was confirmed by their ability to identify 100% toxin-like anemone polypeptides in the reference polypeptide database. The employment of novel motifs for the search of polypeptide toxins in *Anemonia viridis *EST dataset allowed us to identify 89 putative toxin precursors. The translated and modified ESTs were scanned using a special algorithm. In addition to direct comparison with the motifs developed, the putative signal peptides were predicted and homology with known structures was examined.

**Conclusions:**

The suggested method may be used to retrieve structures of interest from the EST databases using simple amino acid sequence motifs as templates. The efficiency of the procedure for directed search of polypeptides is higher than that of most currently used methods. Analysis of 39939 ESTs of sea anemone *Anemonia viridis *resulted in identification of five protein precursors of earlier described toxins, discovery of 43 novel polypeptide toxins, and prediction of 39 putative polypeptide toxin sequences. In addition, two precursors of novel peptides presumably displaying neuronal function were disclosed.

## Background

Expressed sequence tag (EST) analysis is widely used in molecular biology. This analysis comprises the transcriptome of a given tissue at a given time. These data are deposited in a specialized resource at the National Center for Biotechnology Information (NCBI) - dbEST [1]. The EST databases are used to address different problems [2-6].

The EST database analysis requires the development of novel methods and software for data processing. The standard procedure includes processing of the biological material, production of clones, construction of libraries, and data analysis, from grouping in contigs to gene annotation and microarray design [7]. Special program modules facilitating different stages of analysis, such as those for preprocessing of data [8-10] and software for combining sequences in contigs and their annotation, have been developed [11-13]. To improve the quality of initial data processing, the results of different scanning methods can be combined from homology search of a nucleotide consensus sequence, homology search of deduced protein sequences and involvement of reference databases of known organisms [14-17].

The strategy of bioinformatics to database analysis remains the same, variety of diverse crude sequences combined by cluster analysis in contigs should be subjected to alignment search tools and function classification by gene ontologies. It gives good results although is not always optimum. Earlier, analysis of the EST database from spider venomous glands showed [18] that the conventional approach including the preprocessing of the original data and formation of contigs decreased the efficiency of identification of rare polypeptide toxins. The recommended search procedure of scanning translated sequences against characteristic toxin structural motifs proved more effective. Another alternative consists in the use of search queries created from the alignment of known proteins families for database screening. Thus, 83 new peptides were found, which were not earlier discovered in the EST databases of different aphid species [19]. A family of new proteins from corals with a Cys-rich beta-defensin motif was identified as well [20].

Identification of short polypeptides in EST datasets is especially challenging since they may be aligned only with highly homologous proteins. They are synthesized as precursors, which are consequently processed into mature polypeptides. The enzymes involved in maturation recognize specific regulatory amino acid motifs, which help to identify precursor proteins in EST databases [18,19,21].

Polypeptide toxins from natural venoms are of considerable scientific and practical interest. They may be used for designing drugs of new generation [22]. Venom of a single spider contains hundreds of polypeptides of similar three-dimensional structure but divergent biological activity. In toxins, the mature peptide domain is highly variable, while the signal peptide and the propeptide domain are conserved [23,24]. The specificity of action on different cellular receptors depends on the unique combination of variable amino acid residues in the toxin molecule. Using a common scaffold, venomous animals actively change amino acid residues in the spatial loops of toxins thus adjusting the structure of a novel toxin molecule to novel receptor types. This array of polypeptide toxins in venoms is called a natural combinatorial library [25-27].

Homologous polypeptides in a combinatorial library may differ by point mutations or deletions of single amino acid residues. During contig formation such mutations may be considered as sequencing errors and can be ignored. Our method is devoid of such limitations. Instead of the whole EST dataset annotation and search for all possible homologous sequences, we suggest to consider the bank as a "black box", from which the necessary information may be recovered. The criterion for selection of necessary sequences in each particular case depends on the aim of the research and the structural characteristics of the proteins of interest.

To make queries in the EST database and to search for structural homology, we suggest to use single residue distribution analysis (SRDA) earlier developed for classification of spider toxins [28]. In this work, we demonstrate the simplicity and efficacy of SRDA for identifying polypeptide toxins in the EST database of sea anemone *Anemonia viridis*.

## Methods

### SRDA

**Figure 1:**
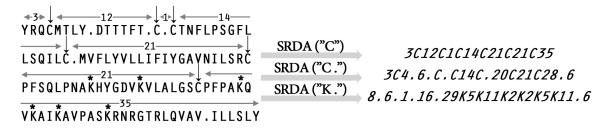
**Conversion of amino acid sequence into a polypeptide pattern using different key residues**. SRDA("C") -conversion by the key Cys residues marked by arrows above the original sequence, the number of amino acids separating the adjacent cysteine residues is also indicated; SRDA("C.") takes into account the location of Cys residues and translational termination symbols denoted by points in the amino acid sequence; ("K.") - conversion by the key Lys residues designated by asterisks and the termination symbols.

In many proteins the position of certain (key) amino acid residues in the polypeptide chain is conserved. The arrangement of these residues may be described by a polypeptide pattern, in which the key residues are separated by numbers corresponding to the number of nonconserved amino acids between the key amino acids (see Figure 1).

For successful analysis, the choice of the key amino acid is of crucial importance. In polypeptide toxins, the structure-forming cysteine residues play this role, for other proteins, some other residues, e.g. lysine, may be as much important (see Figure 1). Sometimes it is necessary to find a specific residues distribution not in the whole protein sequences, but in the most conserved or other interesting sequence fragments. It is advised to start key residue mining in training data sets of limited size. Several amino acids in the polypeptide sequence may be selected for polypeptide pattern construction; however, in this case, the polypeptide pattern will be more complicated. If more than three key amino acid residues are chosen, analysis of their arrangement becomes too complicated. It is necessary to know the position of breaks in the amino acid sequences corresponding to stop codons in protein-coding genes. Figure 1 clearly demonstrates that the distribution of Cys residues in the sequence analyzed by SRDA ("C") differs considerably from that of SRDA ("C.") taking into account termination symbols. For scanning *A. viridis *EST database, the position of termination codons was always taken into consideration.

**Figure 2:**
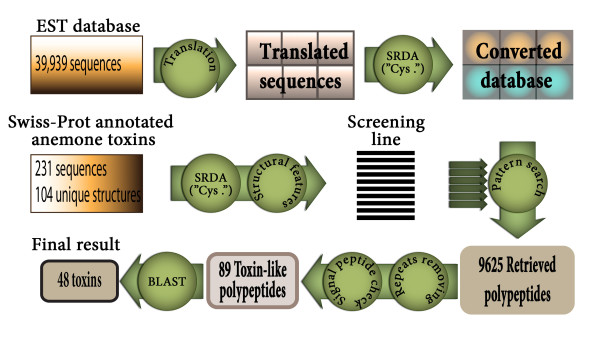
**Flowchart of the analysis pipeline of *A. viridis *ESTs**.

The flowchart of the analysis is presented in Figure 2. The EST database sequences were translated in six frames prior to search, whereupon the deduced amino acid sequences were converted into polypeptide pattern. The SRDA procedure with key cysteine residues and the termination codons was used. The converted database, which contained only identifiers and six associated simplified structure variants (polypeptide patterns), formed the basis for retrieval of novel polypeptide toxins.

To search for sequences of interest, a correctly formulated query is necessary. Queries also in pattern format (screening line in Figure 2) were based on amino acid sequences of anemone toxins after analysis of homology between their simplified structures.

At subsequent stages, from the converted database, amino acid sequences that satisfy each query were selected. Using the identifier, the necessary clones and open reading frames in the original EST database were correlated. As a result, a set of amino acid sequences was formed. Identical sequences, namely identical mature peptide domains without taking into account variations in the signal peptide and propeptide regions, were excluded from analysis. To identify the mature peptide domain, an earlier developed algorithm was used [21,29]. The anemone toxins are secreted polypeptides; therefore only sequences with signal peptides were selected. Signal peptide cleavage sites were detected using both neural networks and Hidden Markov Models trained on eukaryotes using the online-tool SignalP http://www.cbs.dtu.dk/services/SignalP[30]. To ensure that the identified structures were new, homology search in the non-redundant protein sequence database by blastp and PSI-BLAST http://blast.ncbi.nlm.nih.gov/Blast was carried out [31].

### Data for analyses

To search for toxin structures, the EST database created for the Mediterranean anemone *A. viridis *was used [32]. The original data containing 39939 ESTs was obtained from the NCBI server and converted in the table format for Microsoft Excel.

To formulate queries, amino acid sequences of anemone toxins using NCBI database were retrieved. 231 amino acid sequences were deposited in the database to February 1, 2010. All precursor sequences were converted into the mature toxin forms; identical and hypothetical sequences were excluded from analysis. Anemone toxin sequences deduced from databases of *A. viridis *were also excluded. The final number of toxin sequences was 104.

The reference database for review of the developed algorithms and queries was formed from amino acid sequences deposited in the NCBI database. To retrieve toxin sequences, the query "toxin" was used. The search was restricted to the Animal Kingdom. As a result, 10903 sequences were retrieved.

### Computation

EST database analysis was performed on a personal computer using an operating system WindowsXP with installed MS Office 2003. Analyzed sequences in FASTA format were exported into the MS Excel editor with security level allowed macro commands execution (see additional file 1). Translation, SRDA and homology search in the converted database were carry out using special functions on VBA language for use in MS Excel (see additional file 2). Multiple alignments of toxin sequences were carried out with MegAlign program (DNASTAR Inc.).

## Results and Discussion

### Anemone toxin motifs

The development of appropriate queries is the most important part of the analysis. Their tolerance determines the accuracy of EST database screening and finally the number of retrieved sequences. 104 retrieved sequences of mature anemone toxins were subjected to SRDA using a number of key amino acid residues. The best results, as suspected, were obtained with structure patterns based on key cysteine residues. The enrichment in cysteine residues is a characteristic feature of many natural toxins, thus making it possible to use cysteine as a key amino acid residue in data conversion.

Toxins are small compact molecules, whose structure is stabilized by several disulphide bonds. The spatial structure of anemone toxins is divergent on the base of their primary structure feature. We chose cysteine as the key residue for SRDA conversion, and all 104 anemone toxin sequences were processed. More than a dozen screening lines encompassing the whole complexity of anemone toxins were calculated from converted data (see additional file 1). Since amino acid sequence patterns were analyzed, the obtained motifs reflect only the distribution of the key cysteine residues and the position of termination signals (see Table 1). The total number of motifs would be higher, if special substitution symbols were not used.

**Table 1:** Pattern motifs for converted structures of sea anemone toxins obtained with SRDA("C.").

	Screening line	Number of seq.	Example (sequence ID)
motif 1	C1C##C6C#CC	44	Cangitoxin (P82803), AETX-1 (P69943)
motif 2	C1C##C9C#CC#.	8	BDS-II (P59084), APETx2 (P61542)
motif 3	C8C#C*C3C#C.	8	kaliseptin (Q9TWG1), ShK (P29187)
motif 4	C8C*C#C*C3C	9	AsKC1 (Q9TWG0), APHC1 (B2G331)
motif 5	C8C#C*C1C#C#.	2	SHTX-5 (B1B5J0), Gigantoxin-1 (BAD01579)
motif 6	CC#C#CC*C1C*C.	2	AETX-2 (P69944)
motif 7	CC1C*C*C*C*C1C#.	1	PA-TX (P09949)
motif 8	CC1C#C5C*C#.	1	Neurotoxin 3 (1ANS)
motif 9	C6C*C*C*C6C#.	2	acrorhagin II (BAE46983)
motif 10	C8C3C#C.	1	Metridin (P11495)
motif 11	C#C#C#C#C#C#C#C#.	2	Acrorhagin-1 (BAE46981)
motif 12	C6C#C#C1C*C1C	3	AvTX-60A (BAD04943), PsTX-60B (P58912)
motif 13	C#C#C#C#.	1	SHTX-1/SHTX-2 (P0C7W7)
motif 0	###.	18	Equinatoxin II (P61915), Cytolysin-3 (Q9U6X1)
motif 14	##C	2	Up-1 (P0C1G1), bandaporin (BAH80315)
motif K	K > = 6 AND C < = 2		
TOTAL		104	

Each motif was developed from a number of toxin sequences; see some examples in the last column. Symbol # in a screening line corresponds to any digital symbol (0-9), while * designates any suitable omission.

Since the specific operator "Like" was employed for mining toxin sequences in the database, to optimize Screening line the following substitution symbols were used:

? - any single symbol,

# - any single digit (0-9),

* - gap in the search line from 0 to any number of symbols.

Since the final goal by query motifs developing was maximum retrieving of sequences from the database, we didn't try to create universal motifs with broad specificity. Conversely, many motifs were developed to ensure search specificity of key residues distribution in patterns. The first four motifs enclose the largest number of known sea anemone toxins and are the most discriminative. For motifs 5-9, we tried to achieve high identification capacity, while motifs 10-13 were made degenerative and partially overlapped earlier developed motifs.

Among anemone toxins, large cysteine-free molecules exhibiting strong cytolytic activity are present. These toxins named cytolysines comprise a heterogeneous group of membrane-active molecules subdivided into several groups on the basis of primary structure homology and similarity of physical and chemical properties [33]. For these molecules, pattern motifs developed to be too simple (0 and 14 in Table 1) and inadequate for analysis. For identification of such possible structures in databank, a novel motif K was generated; it combined two search parameters: the presence of not more than 2 cysteine residues at SRDA ("C.") and not less than 6 lysine residues at SRDA ("K.").

To check the potential of the developed pattern motifs, the efficiency of retrieval for toxin-like sequences from the reference animal toxin database was determined. Since amino acid sequences of anemone toxins were used as queries, we expected that all anemone toxins would be identified. Due to a specificity of the reference database syntax, the termination symbols in the motifs were eliminated prior to analysis. Table 2 shows the total number of identified sequences, the number of toxins of anemones and coelenterates, as well as the number of toxins in other groups of animals.

**Table 2:** Toxins retrieved from the reference database using pattern motifs.

	total	anemone	Coelenterate	Other taxons	Motif specificity to anemone seq.
motif 1	135	131	131	4	97%
motif 2	15	15	15	0	100%
motif 3	273	20	24	249	7%
motif 4	833	20	36	797	2%
motif 5	46	6	6	40	13%
motif 6	22	2	2	20	9%
motif 7	9	1	1	8	11%
motif 8	5	3	3	2	60%
motif 9	1133	23	37	1096	2%
motif 10	168	6	6	162	4%
motif 11	155	4	5	150	3%
motif 12	48	7	7	41	15%
motif 13	2634	49	70	2564	2%
distinct	7109	154	245	6864	

In the database studied with a total of 13 motifs, we were unable to identify 154 sequences of anemone toxins from 374 available, 108 of which belonged to predicted structures or sequence fragments, and the remaining 46 sequences referred to cytotoxins (motif K).

As shown in Table 2, motif specificity varies considerably that was already mentioned during motif construction. For instance, only motifs 1 and 2 proved specific to anemone toxins. Motifs 3 and 4, early expected to be specific to sea anemones toxin, were also found in toxins of other animals, mainly nematodes and snakes. Although motif 8 was rarest it was found for a spider toxin, a conotoxin and an anemone toxin, therefore it also could not be considered specific.

### Data retrieval from EST database

**Figure 3:**
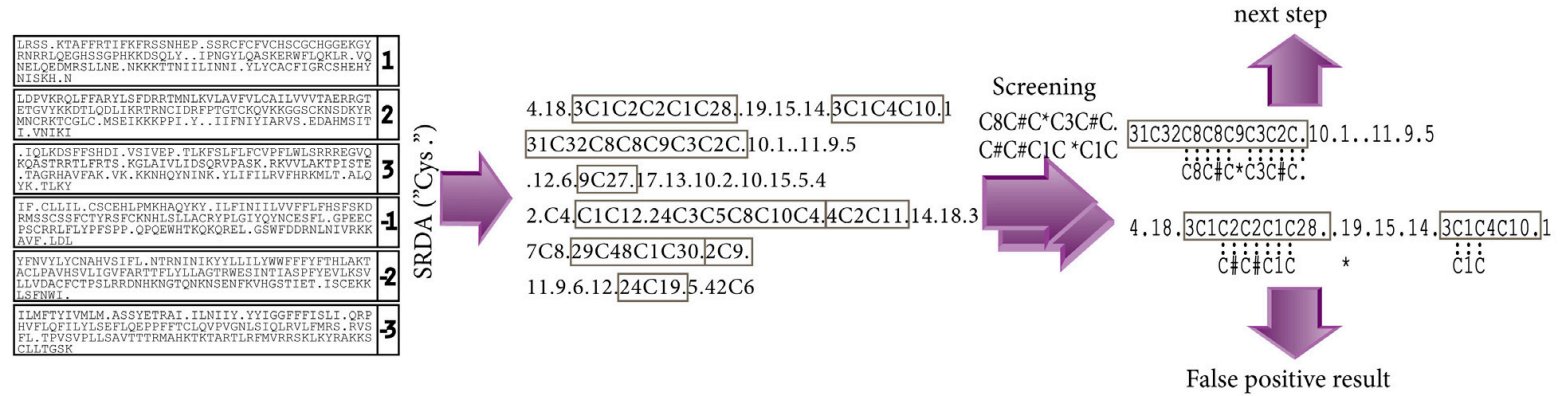
**Pattern search limitation**. Six translated frame should be screened by selected motifs. Sequence fragments between translations stops, in which hit search allowed, are boxed. Identity search for fragment to pattern is permitted inside single fragment and restricted by a multiple fragments implication.

To decrease the number of false positive results during converted database screening, the limitations on the search parameters were imposed. The identity to the screening line was searched only on long fragments started from the beginning or, after any termination symbols and ending by another termination symbol (see Figure 3). If the fragment did not end by the termination symbol, it was rejected as partially identified. The screening analysis was performed on each fragment separately thus a pattern motif must to match completely in the extent of analyzed fragment. This approach considerably decreased the number of false positive results, since it excluded hits with sequences containing internal stop codons (an example of false hit is given in Figure 3).

Each query compared to converted databank resulted dozens sequences in the EST database (see Table 3). As exception, for the most degenerate motif 13 more than 5000 hits were found. Almost all of them matched with sequences in wrong reading frames. This phenomenon was also observed with some other motifs. The obtained false sequences were eliminated at the stage of signal peptide identification. So, it was shown that all sequences retrieved with motifs 6, 7, 8, 10, 12 and most part with motif 13 were false.

**Table 3:** Results obtained from *A. viridis *EST database at each stage of analysis.

	EST retrieved	Nr clones SignalP approved	blastp approved	Known structures found
motif 1	7	4	4	3
motif 2	51	13	13	1
motif 3	162	11	5	0
motif 4	211	16	16	3*
motif 5	26	2	2	-
motif 6	2	0	-	-
motif 7	10	0	-	-
motif 8	8	0	-	-
motif 9	59	2	2	-
motif 10	19	0	-	-
motif 11	81	5	-	-
motif 12	20	0	-	-
motif 13	5466	11	-	-
motif K	133	25	-	-
TOTAL	6222	89	42	7

The total number of sequences found in the database by pattern search designated as "EST retrieved". The number of Non-redundant (Nr) mature sequences keeping signal peptide for secretion designated as "SignalP approved". BlastP approved sequences by blastp and PSI-BLAST algorithm shown identity to anemone toxins, and the number of 100% homologues structures are in the last column.

* including truncated and long variants

In deduced amino acid sequences, the mature peptide chain was determined using a maturation algorithm [21,29], and repetitious mature sequences were discarded. Finally 89 unique secreted sequences possess homology to anemone polypeptide toxins were discovered in *A. viridis *database (see Table 3). Duplicated clones were not numerous; two most abundant sequences revealed with motifs 3 and K were repeated in the database 103 and 58 times, respectively. Detailed information on the correspondence of the deduced polypeptides to the EST nucleotide sequences is given in an additional file 3. Deduced polypeptides were compared on the next processing stage with protein databank resulting in determination of 7 known toxins.

### Polypeptide toxins of A. viridis

The sea anemone *A. viridis *earlier described as *Anemonia sulcata *is an extensively studied Mediterranean species [34-37]. More than 20 polypeptide toxins of different structure and function have been isolated from this species. They include potassium channel blockers, such as kalicludines, kaliseptine, blood depressing substance (BDS) [38,39], neurotoxins effectively blocking sodium channels [40], and Kunitz-type inhibitors of proteolytic enzymes [41,42].

**Figure 4:**
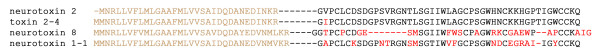
**Alignment of polypeptide structures retrieved with motif 1**. Mature polypeptides are shown in black; signal peptides and propeptide domains are in light brown. Amino acids that differ from the first sequence of the group are shown in red.

Using motif 1, we derive four full-length precursors (see Figure 4), three of which completely coincided with earlier described toxins, sodium channel blockers namely neurotoxin 2, toxin 2-1 and neurotoxin 8. The forth polypeptide named neurotoxin 1-1 had only two substitutions as compared to earlier described neurotoxin 1.

**Figure 5:**
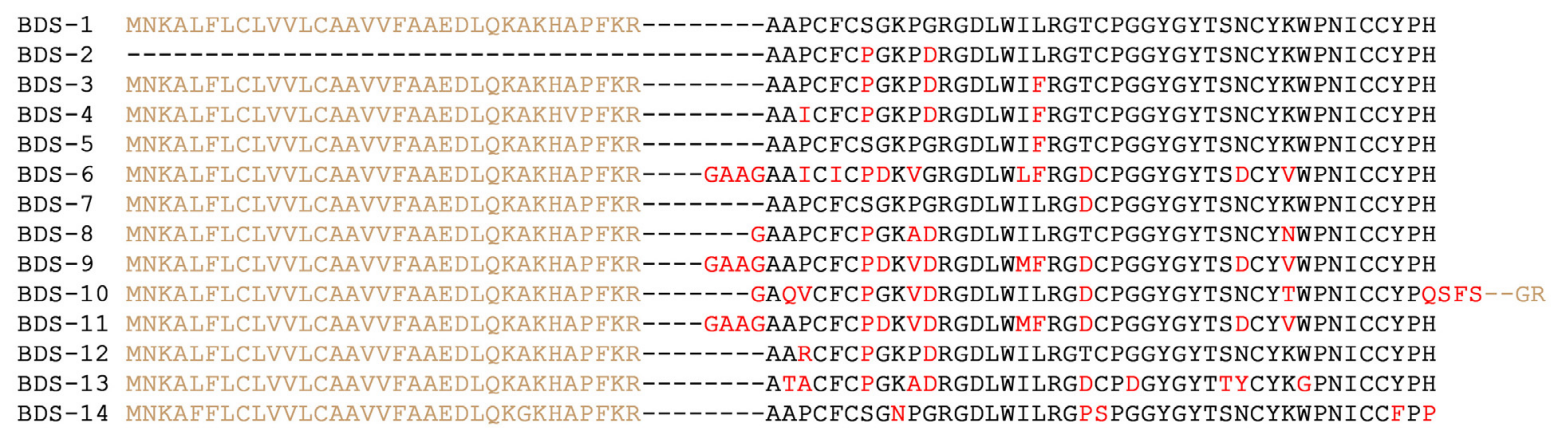
**Alignment of sequences retrieved with motif 2**. Polypeptide toxin BDS-2 (P59084) was not retrieved and shown as structural family member. Mature polypeptides are shown in black, while signal peptides and propeptide domains of precursors are given in light brown; amino acids that differ from the first sequence in the group are shown in red.

The precursor of BDS-1 toxin interacting with the rapidly inactivating Kv_3.4 _channel [39] and 12 homologues of it were discovered in the database with motif 2 (see precursor sequences in Figure 5). All members of the structural family were numbered from 3 to 14. The most abundant among them was the BDS-1 precursor (15 sequences in the EST database). The remaining less represented sequences comprised homologues, which formed the anemone polypeptide toxin combinatorial library.

**Figure 6:**
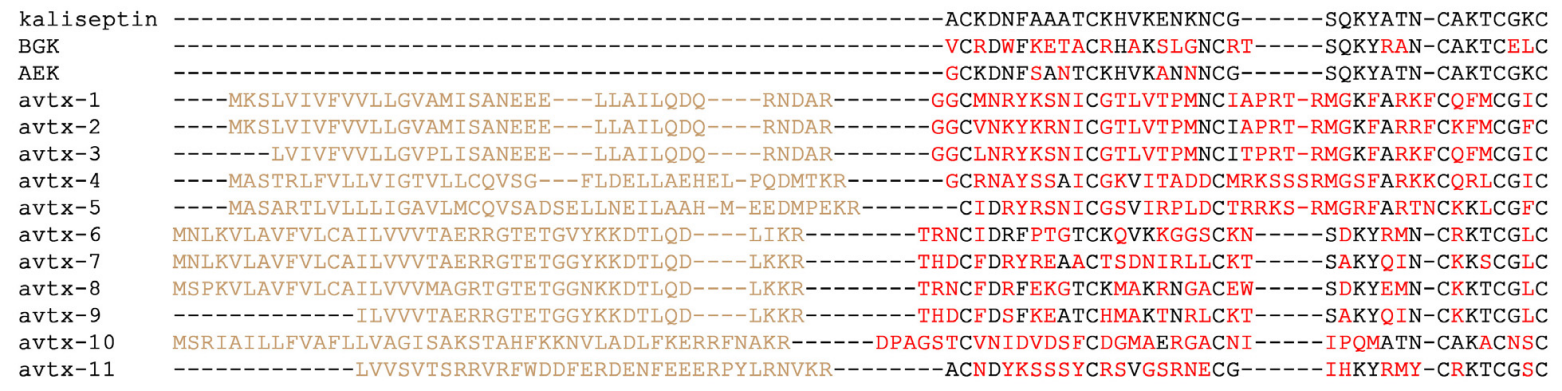
**Alignment of polypeptide structures retrieved using motif 3 vs. potassium channel inhibitors: kaliseptin (Q9TWG1), Bgk (P29186), and Aek (P81897)**. Mature polypeptides are shown in black, signal and propeptides are in light brown; amino acids that differ from kaliseptin sequence are shown in red.

Another known potassium channel blocker kaliseptin [38] was not found in the library, however 11 similar polypeptides using motif 3 as a query (avtx-1 - avtx-11) were identified (see Figure 6). This group displays the lowest similarity to known toxins (see additional file 3), therefore it is possible to assume that they do not act on potassium channels, but exhibit some other still unknown functions. The protein precursor avtx-1 is the most abundant of all structures discovered, we found 103 identical sequences that suggest high expression level and functional significance of the encoded polypeptide.

**Figure 7:**
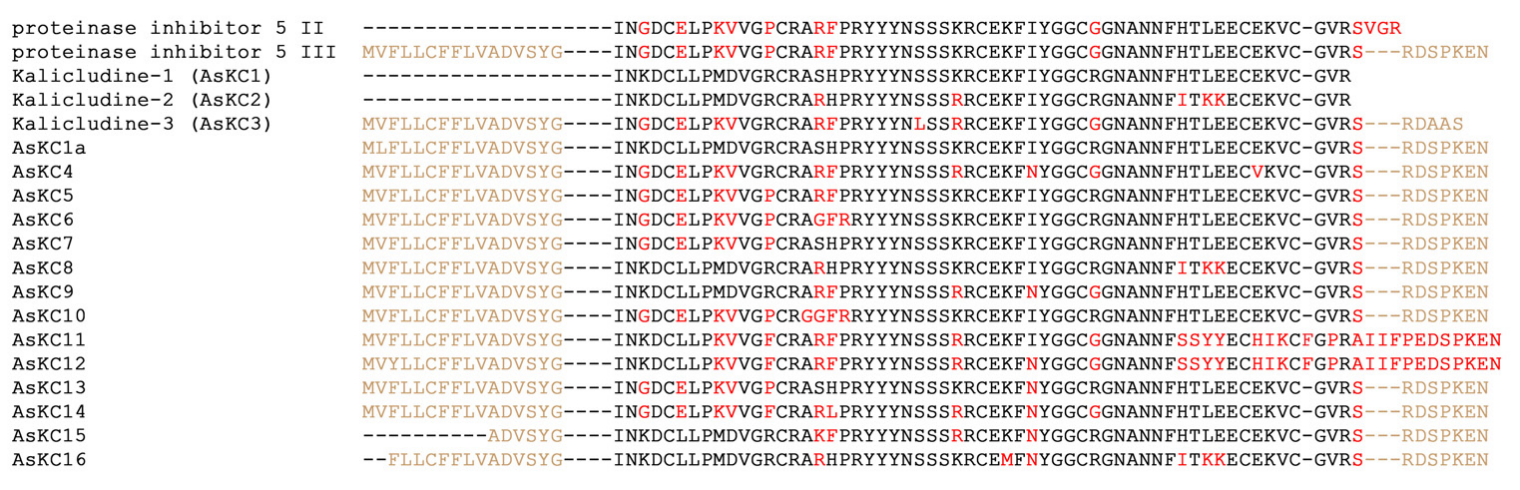
**Alignment of polypeptide structures retrieved with motif 4 vs. BPTI/Kunitz family of serine proteinase inhibitors and toxins (P10280, Q9TWG0, Q9TWF9, Q9TWF8)**. Mature polypeptides are shown in black, while signal peptides and propeptide domains are given in light brown; amino acids that differ from the kalicludine-1 sequence are shown in red.

The Kunitz-type polypeptides were retrieved using motif 4 (see Figure 7). The Kunitz-type scaffold is found not only in inhibitors of proteolytic enzymes but in toxins as well, for example in kalicludines. Some other polypeptides with antifungal and antimicrobial activities and those showing analgesic properties adopt the same scaffold [5,38,42,43]. In this group, the most represented sequences corresponded to the earlier described kalicludine-3 and to a new polypeptide kalicludine-4 (AsKC4). Another less abundant sequence AsKC1a had an additional residue at the *C*-terminus compared to kalicludine-1. Conversely, a novel homologue of a known proteinase inhibitor 5 II named proteinase inhibitor 5 III, which was *C*-terminally truncated by three amino acid residues, was discovered in the database. Other members of the family due to high homology to kalicludines were designated AsKC4-AsKC16.

**Figure 8:**
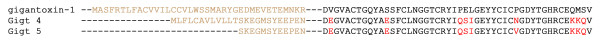
**Comparison of sequences retrieved using motif 5 with gigantoxin-1 precursor (Q76CA1)**. Mature polypeptides are shown in black; signal peptides and propeptide domains are in light brown; amino acids that differ from the sequence of gigantoxin-1 are given in red.

Neurotoxins 3, 7, 9 and 10 reported earlier in anemones [37,42] correlate with 6, 7 and 8 pattern structural motifs, but the relevant sequences were not found in the EST database. Several polypeptides were retrieved with motif 5. Two novel structures Gig 4 and Gig 5 showed high sequence homology to gigantoxin I from another sea anemone species *Stichodactyla gigantean *[44] (see Figure 8). Gigantoxin I is a weak paralytic toxin capable of binding to EGF receptor. However sequence alignment presented in Figure 8 shows that *A. viridis *polypeptides may exhibit different functions. This follows from nonconserved substitutions in the polypeptide chain: V→E, S→E, and QM→KK, which considerably change the charge of the molecule. It has been suggested that generation of toxins with novel functions was accompanied by replacement of functionally important amino acid residues, while the structural fold of the molecule was preserved (this is illustrated by sequences in Figure 8).

**Figure 9:**
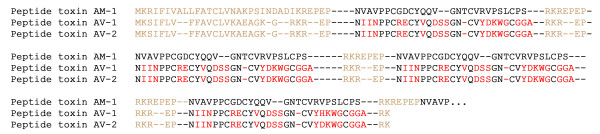
**Alignment of polypeptide sequences retrieved with motif 9 vs. *N*-terminal part of a large precursor of AM-1 toxins (P69929)**. Mature polypeptides are shown in black, while signal peptides and propeptide domains are in light brown; amino acids in AV-toxins that differ from the sequence of AM-1 toxin precursor are given in red.

Two interesting precursors of toxins AV-1 and AV-2 were discovered with motif 9 (see Figure 9). Several polypeptides encoded in a single precursor displayed homology to Am-1 toxins from the sea anemone *Antheopsis maculata *[45]. During maturation, the precursor protein Am-1 is cleaved at the sites of limited proteolysis leading to the production of six active components. In the newly discovered sequences, the number of generated active polypeptides is only four, however the specific amino acid residues involved in a proteolytic cleavage of precursor are identical. For anemone *A. viridis*, the complex structure of the polypeptide toxin precursor has not been described before this work.

Thirty nine sequences were retrieved from the EST database using motifs 11, 13 and K. All of them are presented in the additional file 4. Homology search with blastp algorithm failed to reveal related sequences, however there structures possess correct signal peptides providing effective secretion. For some sequences, the sites of limited proteolysis and the location of the mature peptide domain may be predicted using earlier developed procedures [21,29]. The sequences identified with motifs 11 and 13 were named toxin-like, however their function remains unknown. In the group of short sequences presents only two structural families other sequences are single (additional file 4 panel A). Homology search showed that two sequences Tox-like av-1 and 5 matched earlier predicted structures. Polypeptides Tox-like av-4, 5 and 6 were repetitious in the EST database (see additional file 3).

We also discovered long cysteine-containing sequences named Tox-like av-9 - Tox-like av-16 (additional file 4 panel B). Their structural peculiarities include a long propeptide fragment followed the signal peptide, which is enriched in negatively charged amino acid residues, and numerous arginine and lysine residues in the mature peptide chain. We assume that propeptide can stabilized precursor's structure by compensating excess positive charge of the mature peptide and prevents premature proteolytic degradation, as was demonstrated for precursors of antimicrobial peptides [46,47]. The presence of a large number of positively charged amino acid residues points to possible cytotoxic functions of these peptides.

Several other cysteine-free cytotoxins enriched in lysine residues, the so-called cytolysin-like sequences, were retrieved from the EST database with motif K (additional file 4 panel C). These sequences were repetitive in the database and formed a homologous family (additional file 3). We suppose that natural venom contains truncated variants of these sequences and suggest that two *C*-terminal fragments of about 40 residues in length represent the putative mature polypeptides.

With motif K, 12 short sequences were retrieved from the database. All of them, except one, grouped in four homologous families. Since their functions remain obscure, they were called 'hypothetical peptides' (additional file 4 panel D).

**Figure 10:**
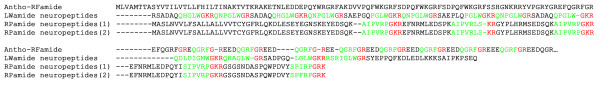
**Amino acid sequence alignment of precursors of LWamide neuropeptides (Q16998), *N*-terminal fragment of Antho-RFamide (Q01133) and novel RPamide neuropeptides retrieved with motif K**. Active neuropeptides are shown in green, identical enzymatic processing sites in precursors are given in red.

In addition, using motif K we discovered two closely related sequences identified as precursors of neuronal peptides (Figure 10). During limited proteolysis, each of them produces five small peptides presumably displaying neuronal activity. Figure 10 shows two examples of known neuropeptide precursors found in anemones, polyps and jelly-fish belonging to the LWamide family, which share the common *C*-terminal sequence Gly-Leu-Trp-NH_2_, or to the RFamide family sharing the *C*-terminal sequence Gly-Arg-Phe-NH_2 _[48,49]. These neuropeptides induce contractions of anemone body wall muscles [50], and in control of metamorphosis in planula larvae of *H. echinata*, LWamides and RFamides work antagonistically [51].

There is no sequence similarity between the precursor proteins presented, however the limited proteolysis motif between generated neuropeptides is similar, and almost all of them keeping a *C*-terminal amidation signal. The localization of the position of the *N*-terminal amino acid residue is problematic; therefore we suggested that active neuropeptides should be consisted of 4-6 amino acid residues. The peptides produced during maturation ended by the sequence Arg-Pro-NH_2 _therefore they were called RPamide neuropeptides.

To summarize, novel polypeptide sequences deduced from *A. viridis *EST database were assembled into several families with members differing by point mutations. This is a common feature of venomous animals, which produce a variety of toxins affecting different targets on the basis of a limited number of sequence patterns. Traditional sequence processing algorithms consider minor sequences as erroneous, but it is not ruled out that these structures are in fact correct. Following proteomic research is necessary to test either possibility.

### The efficiency of the method developed: a comparative study

The SRDA efficiency compared to grouping nucleotide sequences in contigs was earlier demonstrated for the EST database of venomous spider glands [18]. Due to the absence of substantial data on amino acid sequences of homologous proteins, the blast search fails to reveal homology with known proteins. This means that some good consensus sequence and the entire contig will be excluded from a consideration. It is exemplified by the data presented in the additional file 3, where for some sequences the homology was not revealed.

It is more reasonable to compare the efficiency of mining polypeptide sequences using SRDA with other methods, which are also operated with amino acid sequence patterns, such as Pfam or GO [52,53]. This checking was done using a set of amino acid sequences of predicted peptides. Eighty nine sequences in FASTA were downloaded in UFO web server [54]. In comparison with SRDA and blastp, assignment of sequences to protein families by UFO was less successful. The results of search are given for each analyzed sequence in the additional file 3 together with blastp data.

A similar approach was applied for retrieval of polypeptides from the rodent EST database using conserved Cys pattern of the transforming growth factor-β (TGF- β) family [55]. A special Motifer search tool with flexible interface of queries was used. Similarly to our algorithm, Motifer operates with sequences translated in several reading frames and takes into consideration the termination signals. One of the weak points of the program was low database scanning speed.

SRDA simplifies the database itself and the search queries, thus considerably simplify the comparing algorithm and consequently increasing the analysis rate. Thus, the search of 12 queries in the reference database of 10489 sequences on a standard desktop PC required 30 sec. We suggest that the simplicity and high rate of analysis make SRDA attractive not only for the study of polypeptide toxins but of other polypeptides as well.

Since some procedures in the analyses are tedious and labor-consuming, it may be useful to combine SRDA with other progressive techniques, for example based on the Hidden Markov Model. A novel consolidated algorithm will enclose best features of all parts to aid a precise and fast technology of EST processing.

## Conclusions

The SRDA of *A. viridis *EST database showed that this method is effective for rapid retrieval of sequences from the bulk of bioinformatics data. The correct formulation of query plays the crucial role in the outcome of database screening and requires small additional study. The key residues, whose arrangement we wish to fix in the polypeptide pattern, should be selected on the basis of their structural or functional significance. The introduction of termination signals considerably decreases false positive results.

Using the procedure developed, we identified both new sequences and sequences showing high homology to already described toxins. For two known toxins, the precursor structures were determined. All retrieved sequences formed families of homologous peptides that differ by single or multiple amino acid substitutions, providing additional evidence for the combinatorial principle of natural venom formation. In addition to 23 earlier reported polypeptide toxins in sea anemone *A. viridis*, we discovered 43 novel sequences. Besides toxins, we also found short peptides with regulatory neuronal function, whose role is still to be investigated, and several groups of toxin-like polypeptides.

Simplification of queries and the database itself reduces the time of analysis as compared to methods based on the search for complete amino acid sequences. The procedure developed may be used for scanning newly generated databases or as a complementation to traditionally used approaches. It is suitable not only for retrieval of polypeptide toxins but for finding any type of amino acid sequences once their structural motifs have been established.

## Authors' contributions

SK participated in the design of the study, VBA macros creation and performed the analysis. EG conceived of the study, and participated in its design, data interpretation and coordination. All authors read and approved the final manuscript.

## Supplementary Material

Additional file 1 **Supplementary Excel table of reduced databank used in the analysis (read only)**. Use Save as command and allow macros execution to reach SRDA and complementary functions in this example. Click here for file

Additional file 2 **Supplementary listing of VBA module**. General function description and how to use section. Start a MS Excel program and change security level for macros to medium. Open any existent file or create a new one (allow macros execution in the file). Change file type of *Add_file 2 SRDA_processing.bas.txt *to *SRDA_processing.bas *and import all functions included in batch via Microsoft Visual Basic editor (File/import file command).
Type in necessary cell "*= function name(" *and drag "fx" button located on the left from cell input line. Argument(s) required for function processing should be put in the opening window. Then copy equation to other cells.
FUNCTIONS DEFINITION
-Function **ShortDo**(*seq*, *excpt*) - is a main function capable to produce converted sequence, where:
*seq *- String variable enclosed amino acid sequence processed by SRDA,
*excpt *- String variable equal to key residues (combination of any single letter coded amino acid(s) with\without termination symbol".") as a solid word.
-Function **Translate**(*seq*, *frame*) - converts nucleotide to amino acids sequence in appropriated frame, where:
*seq *- String variable enclosed sequence for translation,
*frame *- Integer variable defined translation frame, acceptable value is 1,2,3,-1,-2,-3 or 0 (by frame = 0 only reverse compliment nucleotide sequence will be created).
-Function **SignalFrom**(*seq*, *limitMet*, *frame*, *format*) - is a function for prediction of acceptable Met residue starting a signal peptide, where:
*seq *- String variable enclosed amino acid sequence for processing,
*limitMet *- Integer variable defined a searching range (from the beginning) of Met residue,
*frame *- Integer variable equal to frame used early by translation (frame range 1-6), this variable is important for calculation a position of first nucleotide started possible signal peptide,
*format *- Integer variable defined output style:
0 - function returns the position of the first nucleotide,
1 - function returns the position of the first Met in the signal peptide,
2 - function returns the position of the last nucleotide in predicted signal peptide,
3 - function returns the position of the last amino acid in predicted signal peptide,
other digit - function returns the best score calculated for the signal peptide.
-Function **TrimSeq**(*seq*, *start*, *finish*) - is a function for partial sequence presentation, where:
*seq *- String variable enclosed nucleotide or amino acid sequence,
*start *- Integer variable defined the first nucleotide (amino acid),
*finish *- Integer variable defined the last nucleotide (amino acid).
-Function **MatureChain**(*seq*, *start*, *frame*, *format*) - is a function for sequence termination search, where:
*seq *- String variable enclosed amino acid sequence,
*start *- Integer variable defined a start position for termination symbol searching,
*frame *- Integer variable equal to frame used early by translation (frame range 1-6), this variable is important for calculation a position of the last nucleotide in termination codon,
*format *- Integer variable defined output style:
0 - function returns the position of the last nucleotide in gene,
1 - function returns the position of a termination symbol,
other digit - function returns a polypeptide sequence from *start *to detected terminus.
-Function **Frame6Check**(*pattern*, *seq1*, *seq2*, *seq3*, *seq4*, *seq5*, *seq6*) - prints a frame number in which analyzed sequence(s) match query, where:
*pattern *- String variable defined any text for matching,
*seq1 *- *seq6 *- String variables enclosed amino acid sequences (or converted sequences) translated in 1 to 6 reading frame. Click here for file

Additional file 3 **Supplementary Table**. Results of *A. viridis *EST database processing. Accession numbers of EST sequences in GenBank are given. Homology to known structures was estimated by UFO and PSI-BLAST. Click here for file

Additional file 4 **Supplementary Figure**. Multiple sequence alignment of toxin-like, cytolysin-like and hypothetical peptides. Removable by maturation predicted domains are given in light brown. Cysteine residues are highlighted yellow, while positively charged residues Lysine and Arginine are shown in blue. (A) short toxin-like polypeptides retrieved with motifs 11 and 13; (B) long toxin-like polypeptides retrieved with motifs 11 and 13; (C) Cytolysin-like polypeptides retrieved with motif K and hemolytic toxin Equinatoxin-2 (P61914); (D) hypothetical polypeptides identified with motif K. Click here for file
