# Supplementary material for: The mining of toxin-like polypeptides from EST database by single residue distribution analysis
Source: BMC Genomics. 2011 Jan 31;12:88. doi: 10.1186/1471-2164-12-88 (PMC3040730; doi:10.1186/1471-2164-12-88)
Supplement: Additional file 3 — Supplementary Table. Results of A. viridis EST database processing. Accession numbers of EST sequences in GenBank are given. Homology to known structures was estimated by UFO and PSI-BLAST. [file 1471-2164-12-88-S3.DOC]

Supplementary Table. Results of *A. viridis* EST database processing. Accession numbers of EST sequences in GenBank are given. Homology to known structures was estimated by UFO and PSI-BLAST.

| № | structure name | screening line used | nucleotide sequence GenBank accession No | % ident. | PSI-BLAST homolog found (accession No) | Pfam detected by UFO server |
| --- | --- | --- | --- | --- | --- | --- |
| 1 | Neurotoxin 2 | motif 1 | FK755129, FK730949 | 100 | Neurotoxin 2 (P01528) | PF00706 (Anenome neurotoxin) |
| 2 | toxin 2-4 | motif 1 | FK740862, FK756631 | 100 | toxin 2-4 (ABW97329) | PF00706 (Anenome neurotoxin) |
| 3 | Neurotoxin 8 | motif 1 | FK749453.1 | 100 | Putative neurotoxin 8 (ABW97350) | No assignments |
| 4 | Neurotoxin 1-1 | motif 1 | FK754096.1, FK728784.1 | 95 | Neurotoxin-1 (P01533) | PF00706 (Anenome neurotoxin) |
| 5 | BDS-1 | motif 2 | FK728690, FK727245, FK731753, FK734674, FK756071, FK756154, FK737141, FK733420, FK754388, FK721790, FK735214, FK755366, FK745427, FK727043, FK752129 | 100 | Antihypertensive protein BDS-1 (P11494) | PF07936 (Antihypertensive protein BDS-I/II) |
| 6 | BDS-3 | motif 2 | FK744472, FK753551, FK738010, FK738867, FK730860, FK734966 | 97 | Antihypertensive protein BDS-2 (P59084) | PF07936 (Antihypertensive protein BDS-I/II) |
| 7 | BDS-4 | motif 2 | FK722457, FK734531, FK756277, FK728850, FK727733, FK721844 | 95 | Antihypertensive protein BDS-2 (P59084) | PF07936 (Antihypertensive protein BDS-I/II) |
| 8 | BDS-5 | motif 2 | FK720902, FK737121, FK725211, FK755577, FK758510 | 97 | Antihypertensive protein BDS-1 (P11494) | PF07936 (Antihypertensive protein BDS-I/II) |
| 9 | BDS-6 | motif 2 | FK754940, FK746962, FK725877, FK744302, FK719896 | 76 | Antihypertensive protein BDS-1 (P11494) | PF07936 (Antihypertensive protein BDS-I/II) |
| 10 | BDS-7 | motif 2 | FK736435, FK729210, FK729786 | 97 | Antihypertensive protein BDS-1 (P11494) | PF07936 (Antihypertensive protein BDS-I/II) |
| 11 | BDS-8 | motif 2 | FK723172 | 95 | Antihypertensive protein BDS-2 (P59084) | PF07936 (Antihypertensive protein BDS-I/II) |
| 12 | BDS-9 | motif 2 | FK721972 | 83 | Antihypertensive protein BDS-2 (P59084) | PF07936 (Antihypertensive protein BDS-I/II) |
| 13 | BDS-10 | motif 2 | FK725608, FK742129 | 86 | Antihypertensive protein BDS-2 (P59084) | PF07936 (Antihypertensive protein BDS-I/II) |
| 14 | BDS-11 | motif 2 | FK740326 | 83 | Antihypertensive protein BDS-2 (P59084) | PF07936 (Antihypertensive protein BDS-I/II) |
| 15 | BDS-12 | motif 2 | FK736010 | 97 | Antihypertensive protein BDS-2 (P59084) | PF07936 (Antihypertensive protein BDS-I/II) |
| 16 | BDS-13 | motif 2 | FK752236 | 81 | Antihypertensive protein BDS-2 (P59084) | PF07936 (Antihypertensive protein BDS-I/II) |
| 17 | BDS-14 | motif 2 | FK745823 | 90 | Antihypertensive protein BDS-1 (P11494) | PF07936 (Antihypertensive protein BDS-I/II) |
| 18 | Avtx-1 | motif 3 | FK751977, FK732494, FK750524, FK741022, FK721874, FK752567, FK758080, FK726322, FK721446, FK753446, FK739257, FK758248, FK746508, FK720062, FK723953, FK734597, FK744639, FK750058, FK757190, FK732348, FK759767, FK723781, FK750192, FK740696, FK742823, FK728701, FK749553, FK745347, FK748493, FK746243, FK729443, FK743336, FK758170, FK735626, FK720416, FK745793, FK732733, FK756065, FK745455, FK753254, FK724552, FK721902, FK743536, FK726731, FK758058, FK738785, FK744947, FK756383, FK720578, FK730319, FK723943, FK747663, FK733018, FK744983, FK729764, FK729371, FK730540, FK753818, FK754615, FK752339, FK751113, FK729015, FK731156, FK744443, FK737601, FK755271, FK744048, FK745918, FK749837, FK720750, FK735421, FK740272, FK724618, FK741758, FK727592, FK747325, FK738012, FK729661, FK752493, FK752873, FK739788, FK724995, FK753784, FK739974, FK746450, FK751944, FK749335, FK739716, FK736884, FK752853, FK754880, FK742774, FK741018, FK751415, FK722217, FK740518, FK738209, FK750120, FK742359, FK741695, FK724071, FK749039, FK754777 | 0 | none | No assignments |
| 19 | Avtx-2 | motif 3 | FK759395, FK758305, FK736798, FK724934, FK728565, FK727150, FK724296, FK749433, FK754641 | 0 | none | No assignments |
| 20 | Avtx-3 | motif 3 | FK734835 | 0 | none | No assignments |
| 21 | Avtx-4 | motif 3 | FK734722, FK744863, FK736739, FK738697, FK735015, FK733787, FK756827, FK753497, FK739449, FK720740, FK735694, FK729391, FK750882 | 0 | none | No assignments |
| 22 | Avtx-5 | motif 3 | FK740713, FK724537 | 0 | none | No assignments |
| 23 | Avtx-6 | motif 3 | FK724096, FK738108 | 52 | Potassium channel toxin kaliseptin (Q9TWG1) | No assignments |
| 24 | Avtx-7 | motif 3 | FK756695, FK756784 | 0 | none | No assignments |
| 25 | Avtx-8 | motif 3 | FK755121, FK749797 | 50 | Potassium channel toxin Bgk (P29186) | No assignments |
| 26 | Avtx-9 | motif 3 | FK728190, FK747613, FK747792 | 55 | Potassium channel toxin Bgk (P29186) | No assignments |
| 27 | Avtx-10 | motif 3 | FK743341 | 42 | hypothetical protein | No assignments |
| 28 | Avtx-11 | motif 3 | FK736704 | 45 | Potassium channel toxin Aek (P81897) | No assignments |
| 29 | proteinase inhibitor 5 III | motif 4 | FK749713, FK721810 | 100 | Kunitz-type proteinase inhibitor 5 II (P10280) | PF00014 (Kunitz/Bovine pancreatic trypsin inhibitor domain) |
| 30 | AsKC1a | motif 4 | FK756625, FK756031, FK727341 | 100 | Kunitz-type proteinase inhibitor kalicludin-1 (Q9TWG0) | PF00014 (Kunitz/Bovine pancreatic trypsin inhibitor domain) |
| 31 | AsKC3 | motif 4 | FK750610, FK757545, FK753043, FK753212, FK750940, FK750360, FK722071, FK756445, FK746764, FK734495, FK737670, FK737461, FK732629, FK719946 | 100 | Kunitz-type proteinase inhibitor kalicludin-3 (Q9TWF8) | PF00014 (Kunitz/Bovine pancreatic trypsin inhibitor domain) |
| 32 | AsKC4 | motif 4 | FK745014, FK753410, FK756159, FK746567, FK742258, FK732222, FK741582, FK749202, FK735686, FK727014, FK733855, FK731921, FK739479, FK723226, FK741361 | 94 | Kunitz-type proteinase inhibitor kalicludin-3 (Q9TWF8) | PF00014 (Kunitz/Bovine pancreatic trypsin inhibitor domain) |
| 33 | AsKC5 | motif 4 | FK731360, FK744399, FK734924, FK727999, FK723059 | 98 | Kunitz-type proteinase inhibitor 5 II (P10280) | PF00014 (Kunitz/Bovine pancreatic trypsin inhibitor domain) |
| 34 | AsKC6 | motif 4 | FK753133, FK749316, FK734320, FK719943 | 94 | Kunitz-type proteinase inhibitor 5 II (P10280) | PF00014 (Kunitz/Bovine pancreatic trypsin inhibitor domain) |
| 35 | AsKC7 | motif 4 | FK740712, FK729058, FK742724 | 94 | Kunitz-type proteinase inhibitor 5 II (P10280) | PF00014 (Kunitz/Bovine pancreatic trypsin inhibitor domain) |
| 36 | AsKC8 | motif 4 | FK722334, FK737063 | 98 | Kunitz-type proteinase inhibitor kalicludin-2 (Q9TWF9) | PF00014 (Kunitz/Bovine pancreatic trypsin inhibitor domain) |
| 37 | AsKC9 | motif 4 | FK731883, FK730353 | 91 | Kunitz-type proteinase inhibitor kalicludin-1 (Q9TWG0) | PF00014 (Kunitz/Bovine pancreatic trypsin inhibitor domain) |
| 38 | AsKC10 | motif 4 | FK752628, FK752050 | 93 | Kunitz-type proteinase inhibitor 5 II (P10280) | PF00014 (Kunitz/Bovine pancreatic trypsin inhibitor domain) |
| 39 | AsKC11 | motif 4 | FK727748, FK757246 | 75 | Kunitz-type proteinase inhibitor 5 II (P10280) | PF00014 (Kunitz/Bovine pancreatic trypsin inhibitor domain) |
| 40 | AsKC12 | motif 4 | FK749190 | 73 | Kunitz-type proteinase inhibitor 5 II (P10280) | PF00014 (Kunitz/Bovine pancreatic trypsin inhibitor domain) |
| 41 | AsKC13 | motif 4 | FK753478 | 93 | Kunitz-type proteinase inhibitor 5 II (P10280) | PF00014 (Kunitz/Bovine pancreatic trypsin inhibitor domain) |
| 42 | AsKC14 | motif 4 | FK758844 | 93 | Kunitz-type proteinase inhibitor kalicludin-3 (Q9TWF8) | PF00014 (Kunitz/Bovine pancreatic trypsin inhibitor domain) |
| 43 | AsKC15 | motif 4 | FK741437, FK741043 | 93 | Kunitz-type proteinase inhibitor kalicludin-1 (Q9TWG0) | PF00014 (Kunitz/Bovine pancreatic trypsin inhibitor domain) |
| 44 | AsKC16 | motif 4 | FK744640 | 94 | Kunitz-type proteinase inhibitor kalicludin-2 (Q9TWF9) | PF00014 (Kunitz/Bovine pancreatic trypsin inhibitor domain) |
| 45 | Gigt 4 | motif 5 | FK735889 | 75 | Gigantoxin-1 (Q76CA1) | PF00008 (EGF-like domain) |
| 46 | Gigt 5 | motif 5 | FK749754 | 75 | Gigantoxin-1 (Q76CA1) | PF00008 (EGF-like domain) |
| 47 | Peptide toxin AV-1 | motif 9 | FK719982 | 36 | Peptide toxins Am-1 (P69929) | No assignments |
| 48 | Peptide toxin AV-2 | motif 9 | FK736087 | 36 | Peptide toxins Am-1 (P69929) | No assignments |
| 49 | Tox-like av-01 | motif 11 | FK727324 | 41 | predicted protein | No assignments |
| 50 | Tox-like av-02 | motif 11 | FK739973 | 0 | none | No assignments |
| 51 | Tox-like av-03 | motif 11 | FK743528 | 0 | none | No assignments |
| 52 | Tox-like av-04 | motif 13 | FK757832, FK747472, FK730402, FK725909, FK748676, FK722246, FK734360, FK744516 | 0 | none | No assignments |
| 53 | Tox-like av-05 | motif 13 | FK740941, FK726455, FK742533, FK755035, FK732773 | 54 | hypothetical protein | No assignments |
| 54 | Tox-like av-06 | motif 13 | FK755819, FK740317 | 0 | none | No assignments |
| 55 | Tox-like av-07 | motif 13 | FK752296 | 0 | none | No assignments |
| 56 | Tox-like av-08 | motif 13 | FK754226 | 0 | none | No assignments |
| 57 | Tox-like av-09 | motif 11 | FK756983, FK753605, FK720791, FK732501, FK731994, FK755505, FK728008, FK752668, FK740628, FK734995, FK730755, FK726044 | 0 | none | No assignments |
| 58 | Tox-like av-10 | motif 11 | FK758729, FK749452 | 33 | predicted protein | No assignments |
| 59 | Tox-like av-11 | motif 13 | FK747728 | 0 | none | No assignments |
| 60 | Tox-like av-12 | motif 13 | FK746828 | 0 | none | No assignments |
| 61 | Tox-like av-13 | motif 13 | FK749403, FK723617 | 0 | none | No assignments |
| 62 | Tox-like av-14 | motif 13 | FK724508 | 0 | none | No assignments |
| 63 | Tox-like av-15 | motif 13 | FK737573 | 30 | predicted protein | No assignments |
| 64 | Tox-like av-16 | motif 13 | FK749245 | 50 | predicted protein | No assignments |
| 65 | Cyt-like av-01 | motif K | FK757733, FK740359, FK734970, FK751303, FK747349, FK759585, FK755783, FK729760, FK751621 | 0 | none | No assignments |
| 66 | Cyt-like av-02 | motif K | FK720686, FK745608, FK751543, FK733809, FK734758, FK744117 | 0 | none | No assignments |
| 67 | Cyt-like av-03 | motif K | FK741578, FK746091, FK737756, FK720484 | 0 | none | No assignments |
| 68 | Cyt-like av-04 | motif K | FK757018, FK747075, FK738566 | 0 | none | No assignments |
| 69 | Cyt-like av-05 | motif K | FK739869, FK758592, FK744970 | 0 | none | No assignments |
| 70 | Cyt-like av-06 | motif K | FK755563, FK744553, FK732483 | 0 | none | No assignments |
| 71 | Cyt-like av-07 | motif K | FK748084, FK722426, FK720267 | 0 | none | No assignments |
| 72 | Cyt-like av-08 | motif K | FK749308, FK759717 | 0 | none | No assignments |
| 73 | Cyt-like av-09 | motif K | FK757975 | 0 | none | No assignments |
| 74 | Cyt-like av-10 | motif K | FK746103 | 0 | none | No assignments |
| 75 | Cyt-like av-11 | motif K | FK725305 | 0 | none | No assignments |
| 76 | hpp av-01 | motif K | FK745453, FK744864, FK732164, FK744950, FK744645, FK731216, FK731378, FK736336, FK745692, FK722672, FK742138, FK747754, FK743757, FK734851, FK739643, FK723623, FK729209, FK721640, FK753916, FK755001, FK729244, FK737485, FK753028, FK745049, FK744530, FK741620, FK726675, FK732641, FK735879, FK753309, FK746449, FK759016, FK756504, FK725073, FK746537, FK742046, FK730381, FK751035, FK752926, FK746694, FK730785, FK741301, FK737148, FK749627, FK742970, FK750214, FK759179, FK726800, FK756947, FK738632, FK742344, FK724559, FK724064, FK720849, FK736431, FK727598, FK748869, FK727016 | 0 | none | No assignments |
| 77 | hpp av-02 | motif K | FK735026, FK736849, FK745099 | 0 | none | No assignments |
| 78 | hpp av-03 | motif K | FK741832 | 0 | none | No assignments |
| 79 | hpp av-04 | motif K | FK752633 | 0 | none | No assignments |
| 80 | hpp av-05 | motif K | FK726784 | 0 | none | No assignments |
| 81 | hpp av-06 | motif K | FK738458, FK736807, FK758888, FK732278 | 0 | none | No assignments |
| 82 | hpp av-07 | motif K | FK735165 | 0 | none | No assignments |
| 83 | hpp av-08 | motif K | FK732466, FK744093 | 0 | none | No assignments |
| 84 | hpp av-09 | motif K | FK728004 | 0 | none | No assignments |
| 85 | hpp av-10 | motif K | FK726997, FK722345 | 0 | none | No assignments |
| 86 | hpp av-11 | motif K | FK722477, FK738199 | 0 | none | No assignments |
| 87 | hpp av-12 | motif K | FK753558, FK732038 | 0 | none | No assignments |
| 88 | RPamide neuropeptides(1) | motif K | FK744635, FK729471 | 0 | none | No assignments |
| 89 | RPamide neuropeptides(2) | motif K | FK720714 | 0 | none | No assignments |
