# Supplementary figures and images for: The mining of toxin-like polypeptides from EST database by single residue distribution analysis
Source: BMC Genomics. 2011 Jan 31;12:88. doi: 10.1186/1471-2164-12-88 (PMC3040730; doi:10.1186/1471-2164-12-88)

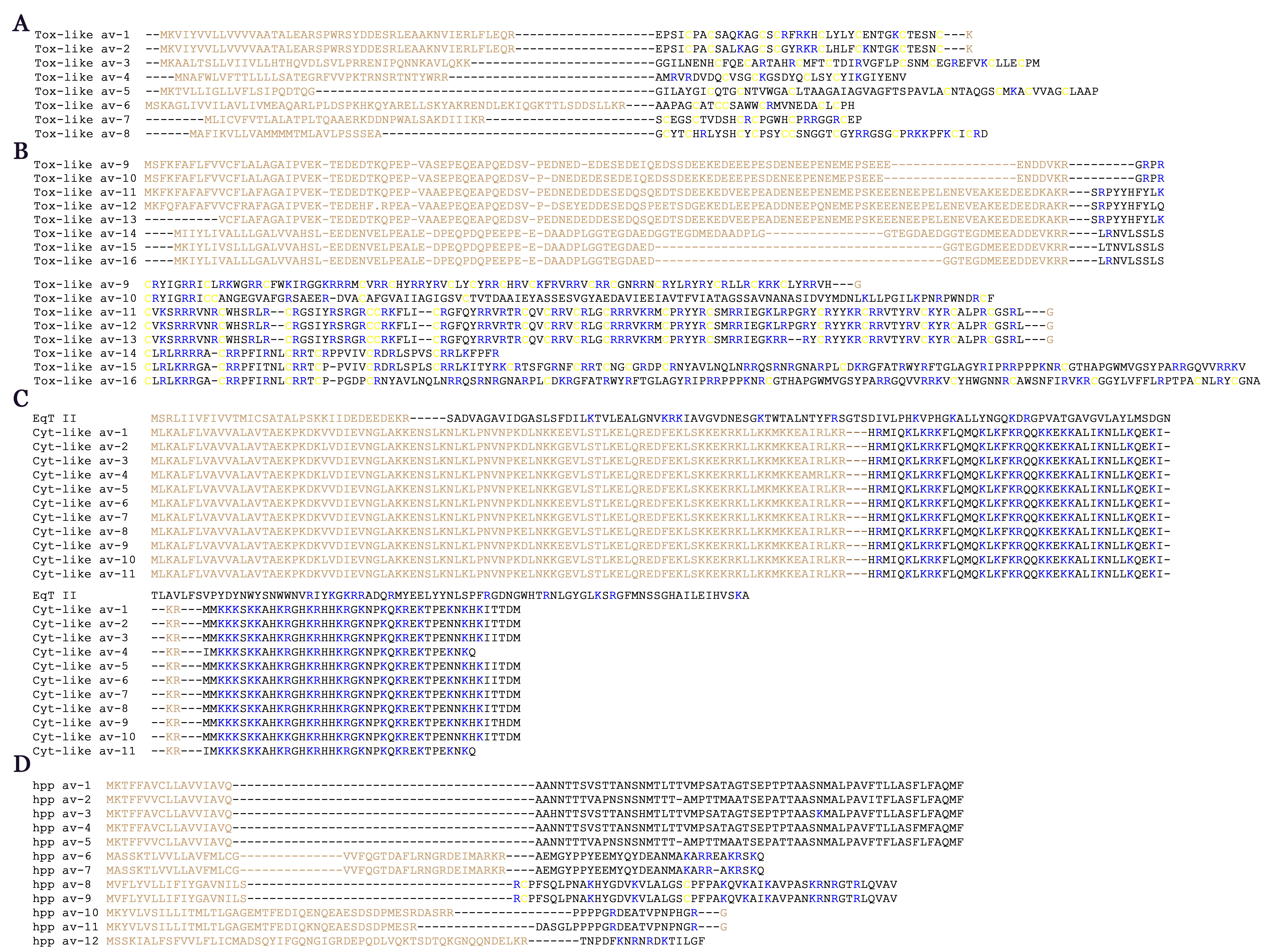

Supplement: Additional file 4 — Supplementary Figure. Multiple sequence alignment of toxin-like, cytolysin-like and hypothetical peptides. Removable by maturation predicted domains are given in light brown. Cysteine residues are highlighted yellow, while positively charged residues Lysine and Arginine are shown in blue. (A) short toxin-like polypeptides retrieved with motifs 11 and 13; (B) long toxin-like polypeptides retrieved with motifs 11 and 13; (C) Cytolysin-like polypeptides retrieved with motif K and hemolytic toxin Equinatoxin-2 (P61914); (D) hypothetical polypeptides identified with motif K. [file 1471-2164-12-88-S4.JPEG]
